# Photorespiratory Metabolism and Its Regulatory Links to Plant Defence Against Pathogens

**DOI:** 10.3390/ijms252212134

**Published:** 2024-11-12

**Authors:** Iwona Ciereszko, Elżbieta Kuźniak

**Affiliations:** 1 Department of Biology and Plant Ecology, Faculty of Biology, University of Bialystok, Ciolkowskiego 1J, 15-245 Bialystok, Poland; 2 Department of Plant Physiology and Biochemistry, Faculty of Biology and Environmental Protection, University of Lodz, Banacha 12/16, 90-237 Lodz, Poland

**Keywords:** chloroplast immune response, infection, mitochondrial enzymes, peroxisome metabolites, photorespiration, plant–pathogen interaction, reactive oxygen species

## Abstract

When plants face biotic stress, the induction of defence responses imposes a massive demand for carbon and energy resources, which could decrease the reserves allocated towards growth. These growth–defence trade-offs have important implications for plant fitness and productivity and influence the outcome of plant–pathogen interactions. Biotic stress strongly affects plant cells’ primary metabolism, including photosynthesis and respiration, the main source of energy and carbon skeletons for plant growth, development, and defence. Although the nature of photosynthetic limitations imposed by pathogens is variable, infection often increases photorespiratory pressure, generating conditions that promote ribulose-1,5-bisphosphate oxygenation, leading to a metabolic shift from assimilation to photorespiration. Photorespiration, the significant metabolic flux following photosynthesis, protects the photosynthetic apparatus from photoinhibition. However, recent studies reveal that its role is far beyond photoprotection. The intermediates of the photorespiratory cycle regulate photosynthesis, and photorespiration interacts with the metabolic pathways of nitrogen and sulphur, shaping the primary metabolism for stress responses. This work aims to present recent insights into the integration of photorespiration within the network of primary metabolism under biotic stress. It also explores the potential implications of regulating photosynthetic–photorespiratory metabolism for plant defence against bacterial and fungal pathogens.

## 1. Introduction

In the natural environment, diseases caused by microbial pathogens compromise plant survival and reproduction. In agriculture, they may lead to a substantial loss in crop yield and downstream impacts on human health [1,2]. The plant immune system, which allows plants to resist attacks from pathogenic microorganisms, is activated following recognising pathogens at the cell surface. This process is mediated by pattern recognition receptors (PRRs) that recognise pathogen-derived elicitors known as pathogen-associated molecular patterns (PAMPs) or endogenous molecules released during pathogen infection. These initiate immune responses constitute pattern-triggered immunity (PTI), consisting of reactive oxygen species (ROS) production, calcium (Ca^2+^) influx at the plasma membrane, defence-related phytohormone biosynthesis, mitogen-activated protein kinase (MAPK) and calcium-dependent protein kinase (CDPK) immune signalling and transcriptional reprogramming of plant cells. PTI confers basal resistance to a broad spectrum of pathogens [3,4]. A secondary line of plant defence called effector-triggered immunity (ETI) is initiated by sensing the pathogen effectors through the intracellular nucleotide-binding/leucine-rich repeat (NLR) receptors. ETI represents a more specialised and robust defence mechanism, often exemplified by the hypersensitive response (HR) [5,6]. Although PTI and ETI are usually considered independent branches of the plant immune system, recent studies showed that effective defence against pathogens relies on the comprehensive interaction between PTI and ETI [7,8].

To defend against pathogens, plants allocate metabolic resources and energy to their defence mechanisms. The activation of the plant immune system often comes at the expense of growth and development, a phenomenon described as the growth–immunity trade-off [9]. This has been confirmed by numerous data on the constitutive expression of resistance to pathogens. For example, in susceptible *Arabidopsis* ecotypes, the expression of the resistance gene *RPM1*, encoding resistance to *Pseudomonas syringae*, led to decreased shoot biomass and seed production in a field trial, indicating that the constitutive induction of defence genes comes with a high metabolic cost [10]. Data from mutants constitutively expressing resistance and growing in the absence of pathogens are also consistent with the hypothesis that the constitutive expression of resistance leads to fitness costs [11]. Moreover, the application of chemical defence elicitors such as benzo(1,2,3)thiadiazole-7-carbotionic acid-S-methyl ester (BTH), an effective systemic-acquired resistance (SAR) inducer in plants, reduced plant growth in the absence of pathogens, mainly when nitrate nitrogen availability was limited [12]. In contrast, when plants grow fast, e.g., subjected to vegetational shading, their susceptibility to biotic stress is often increased [13]. The equilibrium between growth and defence has essential ecological consequences in wild plants because both processes must be balanced to ensure survival and reproduction. Concerning crops, which have often been bred to maximise traits related to growth and productivity, it is vital for food security [13,14,15]. Therefore, understanding the endogenous mechanisms underlying the growth–defence trade-offs could support future crop breeding strategies to overcome these limitations while enhancing disease resistance. Possible systemic growth suppression during an increased state of immunity has been suggested to result from alterations in the diversion of energy and carbon skeletons to biosynthesis pathways of defence mediators, proteins, and metabolites [13], and the physical loss of photosynthetically active tissues due to infection symptom development is not a prerequisite for growth delays.

In photosynthetically active plants, photorespiration links photosynthetic carbon assimilation with other processes, such as nitrogen and sulphur assimilation and the tricarboxylic acid cycle (TCA) [16,17]. This integration may be crucial for allocating metabolic resources and energy to induce defence against pathogens [18]. Moreover, environmental changes, such as temperature and CO_2_ concentration increases, modulate photorespiration and strongly influence pathogens and plant–pathogen interactions [19]. For example, the predicted climate-change-related temperature increase may extend the geographic range of some pathogens currently limited by temperature. The establishment of migrant virulent pathogens, as climatic conditions become more favourable to them, and the emergence of new disease epidemics in a warming climate have become a significant threat concerning plant interactions with pathogens. Moreover, elevated temperature also modulates plant immune responses, e.g., those mediated by salicylic acid (SA), a central defence phytohormone [20]. Simultaneously, the predicted increase in the atmospheric CO_2_ concentration may decrease the photorespiratory rate [21]. As photorespiration interacts with several primary metabolic pathways, including nitrogen and sulphur assimilation, the decrease in the photorespiratory rate resulting from the CO_2_ concentration increase might threaten plant growth by reducing the synthesis of essential metabolites. Thus, its suppression may affect the plant defence potential under a changing climate.

In this context, photorespiration, its integration within the primary metabolism, and its contribution to plant immune response networks [22] are particularly important for further efforts to design new strategies to engineer the plant metabolism for improved crops with better growth, yields, and pathogen resistance. Thus, our review explores the role of photorespiration and the metabolic regulatory interactions among chloroplasts, mitochondria, and peroxisomes in plant defence against bacterial and fungal pathogens.

## 2. Photorespiration and Its Role in the Network of Plant Cell Metabolism

**Figure 1:**
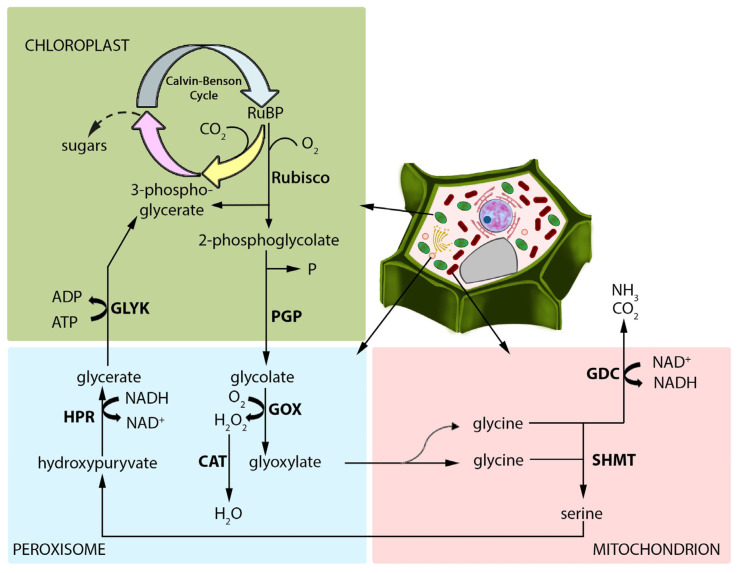
Photorespiratory pathway overview (simplified scheme). The photorespiration cycle occurs in the cell’s three main compartments: chloroplasts, peroxisomes and mitochondria; these organelles are often located close to each other, which allows for the efficient exchange of metabolites during the cycle. The photorespiration cycle is also called C2, oxidative photosynthetic carbon pathway. Gas exchange associated with photorespiration is the uptake of oxygen and the release of CO_2_ (in light) and NH_3_. The first reaction that starts photorespiration occurs in the chloroplast: the oxygenation of the ribulose-1,5-bisphosphate (RuBP) catalysed by the enzyme RuBP carboxylase/oxygenase (Rubisco); products of this reaction are 3-phosphoglycerate and 2-phosphoglycolate. The enzyme Rubisco also catalyzes the assimilation of carbon dioxide (to which it has a greater affinity) to RuBP, initiating the Calvin–Benson cycle reaction of photosynthesis in the chloroplast stroma. Carbon dioxide or oxygen, substrates for Rubisco, enter the leaves through open stomata and diffuse into the chloroplasts. The oxygenation product, 2-phosphoglycolate, is dephosphorylated by phosphatase (PGP) into glycolate, which is transported to the following organelle, the peroxisome (chloroplastic glycolate/glycerate translocator in the inner membrane is known to be involved). Glycolate is oxidised to glyoxylate and H_2_O_2_ thanks to glycolate oxidase (GOX) activity (or glycolate dehydrogenase–in green algae). Glyoxylate is then converted to glycine in the peroxisomes by aminotransferases: glutamate: glyoxylate aminotransferase (GGAT) and serine: glyoxylate aminotransferase (SGAT). Glycine is then transported to the mitochondrion, where two molecules are converted to serine in reactions catalysed by the multienzyme glycine decarboxylase (GDC, a complex that consists of P-protein, T-protein and H-, L-proteins), and serine hydroxymethyltransferase (SHMT); CO_2_ and NH_3_ are released in those reactions. Serine is exported to peroxisome, where the action of aminotransferase SGAT converts it into hydroxypyruvate, and next, the enzyme hydroxypyruvate reductase (HPR) catalyses the reduction of hydroxypyruvate to glycerate. Glycerate is transported to the chloroplast, where the phosphorylation reaction is catalysed by glycerate-3-kinase (GLYK), and 3-phosphoglycerate is produced, ready to be involved in the Calvin–Benson cycle. Abbreviations: CAT—enzyme catalase; GDC—glycine decarboxylase; GLYK—glycerate-3-kinase; GOX—glycolate oxidase; HPR—3-hydroxy pyruvate reductase; PGP—2-phosphoglycolate phosphatase; Rubisco—ribulose-1,5-bisphosphate carboxylase/oxygenase; RuBP—ribulose-1,5-bisphosphate; SHMT—serine hydroxy-methyltransferase (enzyme that catalyses the reversible interconversion of serine and glycine).

Photorespiration, a high-flux pathway orchestrated across chloroplasts, peroxisomes, and mitochondria, plays a fundamental role in plant metabolism (Figure 1).

Photorespiration has long been considered a wasteful process that potentially decreases photosynthetic carbon assimilation, plant growth, and productivity due to a loss of CO_2_ and energy. Consequently, reducing photorespiratory losses has been proposed as a valuable target for crop improvement [21]. Recent studies demonstrated that optimising photorespiration may improve the efficacy of C_3_ photosynthesis and growth [23,24]. They also indicated the importance of photorespiration in regulating carbon metabolism and plant responses to stresses [18,22,25,26,27]. It is assumed that the reprogramming of plant metabolism is an essential aspect of plant–pathogen interactions, and three main aspects of pathogen infection effects on plant primary metabolism have emerged: (1) the energetic and metabolic costs of the induced defence; (2) changed photosynthetic activity due to the pathogen-induced cellular damage in the photosynthetic tissues; (3) the attempted pathogen manipulation of the plant metabolism for nutritional purposes. Therefore, rapid adaptive metabolic flux is a prerequisite to counteract pathogen-induced biotic stress.

Cellular organelles where photorespiration occurs are often located close to each other, facilitating the exchange of metabolites (Figure 1). In addition, it has recently been found that they develop tubular extensions of membranes called stromules (for chloroplasts), peroxules (for peroxisomes), or matrixules (for mitochondria) [28]. Stromules and their role in stress are becoming better characterised, while other extensions and their importance in plant cells are at an early research stage. Through photorespiration, the metabolic interactions between chloroplasts, mitochondria, and peroxisomes contribute to defence against pathogens [29,30,31]. Photorespiration can provide signals, substrates, and energy for immune responses. Although the data on the activity of photorespiratory genes and enzymes in the infected plants are ambiguous [32,33] and depend on the specific plant–pathogen interaction, the immune system appears to take advantage of photorespiration, as we present in the following sections of this review.

## 3. Photosynthesis and Photorespiration in Plants Under Biotic Stress

Photosynthesis and photorespiration are highly sensitive to stress, and many intermediates in these pathways respond to biotic stress. Infected plants suffer from reduced availability of CO_2_ for photosynthesis because both leaf diseases and resistance responses can cause stomata to close [34,35]. Stomatal closure, initiated by sensing PAMPs, is a typical response to prevent pathogen entry, known as stomatal immunity [36]. This process is associated with increased plant defence phytohormones, such as abscisic acid (ABA), synthesised in guard cells, and SA [37,38]. Stomata–pathogen interactions are dynamic, and successful pathogens open stomata by secreting phytotoxins or effectors to enter the leaf. The specific close–open–close–open pattern in plant stomata–pathogen interactions can be observed [36]. After entry, some pathogens induce stomatal closure; however, resistant plants tend to re-open stomata through defence signalling to starve pathogens of water and nutrients.

A non-uniform closure of the stomata and patchiness of the photosynthetic CO_2_ assimilation can also occur along with the progress of the disease and the formation of necrotic or chlorotic lesions [39]. When the stomata are closed, O_2_ is produced by Photosystem II and its concentration increases. At the same time, CO_2_ is consumed by carboxylation, and, thus, its concentration decreases. Therefore, the CO_2_ to O_2_ ratio inside the leaf decreases, which promotes the oxygenation of Rubisco [21]. Consequently, the intracellular CO_2_ is lowered, transpiration decreases, and the temperature rises, favouring photorespiration and further ROS overproduction [37,40]. Stomatal closure benefits plants under pathogen invasion, forming the first line of defence [41], and it also triggers long-term effects related to decreased photosynthetic carbon assimilation and increased photorespiratory activity [38].

Like most environmental stress factors that limit gas exchange, pathogen infection promotes increased excitation energy, thus influencing light energy capture, photosynthetic electron transport, and carbon fixation through photosynthesis [42]. During infection, the ratios of Rubisco-catalysed carboxylation and oxygenation can deviate from the current homeostasis, and metabolism needs to be reprogrammed to adjust the metabolic processes to a new state of homeostasis. In an unstable metabolic state of photosynthesis, an imbalance of ATP/ADP and NADPH/NADP ratios can lead to ROS overproduction and tissue damage [40,43]. However, photorespiration provides photoprotection by dissipating excess excitation energy in the absence of sufficient CO_2_ as an electron acceptor and reducing ROS generation, as well as contributing to redox homeostasis during biotic challenge [44,45]. Photorespiratory reactions can dissipate excess energy, reducing equivalents either directly (using ATP, NAD(P)H and reduced ferredoxin) or indirectly (via alternative oxidase (AOX) and providing an internal CO_2_ pool) [43,46,47]. Ferredoxin, being the uppermost electron acceptor in the chloroplast electron transport chain, plays a crucial role in determining the redox status of downstream reductants, such as NADPH and thioredoxins. NADPH, produced in chloroplasts by ferredoxin-NADP^+^ reductase, is used in defence-related anabolic processes and the regeneration of antioxidants by NADPH-dependent enzymes [48]. Furthermore, ferredoxin and NADPH are involved in redox signalling through ferredoxin- and NADPH-dependent thioredoxin reductases in chloroplasts. These processes are also crucial for maintaining the redox balance mediated by the ascorbate-glutathione cycle, and they play a role in regulating disease resistance [29,40,49]. Moreover, the enzyme named plastid terminal oxidase (PTOX), a plastoquinone-oxygen oxidoreductase localised in chloroplast membranes, is probably acting as a photoprotective valve for photosynthesis and protects photosystems against oxidative damage under conditions where reducing power is being produced in excess [50,51,52]. It would be interesting to investigate and understand the function of this enzyme under biotic stress conditions in plants.

## 4. Photorespiratory ROS in Plant Defence Against Pathogens

Photorespiration is a significant source of H_2_O_2_ in photosynthetic cells (Figure 1). It has been estimated that 70% of the total H_2_O_2_ may come from photorespiration, and glycolate oxidation, catalysed by glycolate oxidase (GOX), is proposed to be the primary source of this signalling molecule in green tissues [53,54]. H_2_O_2_ plays multiple roles during pathogen attack, including cell wall strengthening and activation of phytoalexin biosynthesis. It can be toxic to pathogens, trigger HR, and is involved in immune signal transduction, resulting in the modulation of gene expression [55,56]. Simultaneously, all photorespiratory organelles have ROS-processing systems that contribute to the cellular redox regulatory mechanism [29]. It is now established that redox signalling is essential for the functioning of the plant immune system and can shape plant–pathogen interactions. Moreover, pathogens hijack plant redox signalling and utilise their antioxidant systems to promote virulence and successful development within the host [57]. Chloroplasts, mitochondria, and peroxisomes are essential organelles for photorespiratory ROS generation and signalling during pathogenesis. Their crosstalk during the infection-induced production of ROS, particularly H_2_O_2_, has been well documented [58,59,60]. As long as apoplastic ROS generation is associated with PAMP-triggered early defence responses, ROS bursts and redox changes in chloroplasts, peroxisomes, and mitochondria have been related to late defence reactions such as ETI and HR [55,58,60,61]. A shift in the cellular redox balance towards an oxidative state also resulted from the successful infection of tomato (*Solanum lycopersicum*) leaf cells by *Botrytis cinerea*. However, the intensity and timing of the redox changes in chloroplasts, mitochondria, and peroxisomes were organelle-specific [29].

From the photorespiratory organelles, chloroplasts have emerged as a central hub in plant immunity. The prominent function of chloroplasts in defence is related to generating pro-defence signals, e.g., ROS, phytohormones, and metabolic intermediates that function in retrograde communication with the nucleus [55]. Chloroplasts harbour essential steps in the biosynthesis of defence phytohormones, SA, jasmonic acid (JA), and ABA. Xanthoxine produced by the xanthophyll pathway is the precursor of ABA, chorismate is the precursor of SA, and oxidised lipids such as linolenic acid are required for JA biosynthesis [62]. Chloroplast H_2_O_2_ plays a central role in regulating the response to SA, a key regulator of biotic stress. Silencing of thylakoid membrane-bound ascorbate peroxidase (APX), a significant H_2_O_2_-scavenging enzyme in chloroplasts, enhanced the level of SA and the response to SA [63]. Moreover, a functional difference between chloroplast and peroxisomal H_2_O_2_ was suggested in regulating the response to SA.

PAMPs and pathogen effectors target chloroplasts. For example, after perceiving PAMP, such as flg22, a peptide PAMP from bacterial flagellin, *Arabidopsis* calcium-dependent protein kinase 16, relocalises from the plasma membrane to chloroplasts to induce the immune response [64]. During *Nicotiana benthamiana* infection with *Phytophthora infestans*, chloroplasts accumulated at pathogen haustoria and altered their morphology through the induction of stromules, which facilitated the chloroplast–chloroplast contact at the pathogen interface. Stromules were induced by flg22 through BRASSINOSTEROID INSENSITIVE 1-ASSOCIATED KINASE 1 (BAK-1)-mediated signalling, and the pathogen effector counteracted this defence response [65]. Upon activation of ETI, *N. benthamiana* chloroplasts establish physical contact with the nucleus through clustering around it and extending stromules. The perinuclear clustering of chloroplasts was suggested to be elicited by H_2_O_2_ [66,67]. Although the biological significance of chloroplast relocalisation and stromule formation upon pathogen recognition remains elusive, these results support a model in which they are tightly linked to innate immune responses.

Pathogens target chloroplasts and disrupt their function to achieve virulence. They secrete effectors that alter host gene expression, including that of the nucleus-encoded chloroplast genes, deliver effectors that enter the chloroplasts and can interact with host chloroplast proteins, and change the localisation of non-chloroplast host proteins (reviewed by [68]). Some published research results indicate several pathogen effectors, which can affect SA synthesis and signalling. Fungal pathogens *Phytophthora sojae* and *Verticillium dahliae* secrete isochorismatases when infecting *Arabidopsis* and cotton to hydrolyse isochorismate, an SA precursor. These enzymatic effectors are required for full pathogenesis. They inhibit SA accumulation and suppress host immunity by redirecting the SA precursor from chloroplasts to the cytosol, where isochorismate is the substrate for isochorismatases [69]. The pathogenic bacteria *P. syringae* adopts other mechanisms, suppressing SA biosynthesis in host cells. Its effector HopI1 targeted to chloroplasts depresses the SA level and suppresses host defence by remodelling the chloroplast thylakoid structure [70].

It has been suggested that plants must suppress their abiotic stress tolerance mechanisms, including chloroplast reactions related to ROS management, to generate immune signals [68]. Pathogens attempt to reactivate these mechanisms, steering the plant to induce an inappropriate defence response and promote infection.

During the non-host interaction of tobacco (*Nicotiana tabacum*) with *Xanthomonas campestris* pv *vesicatoria*, the chloroplast redox status modulated the expression patterns of genes associated with plant defence responses, such as the synthesis of PR proteins, cell wall reinforcement, signal transduction, and antioxidant metabolism [71]. Moreover, the changes in chloroplast redox status induced by pathogens may also contribute to the retrograde signalling from the chloroplast to the nucleus [59,72]. Hydrogen peroxide produced by chloroplasts transmits the stress signal to the nucleus to regulate gene expression through modifying redox-sensitive transcription factors, controlling the cellular redox status or reacting with protein thiol groups [73]. The Photosystem I acceptor side was suggested to produce sufficient H_2_O_2_ to participate in retrograde signalling and impact the redox state of the cellular ROS processing system [74].

ROS generated in chloroplasts due to pathogen-induced dysfunction in the photosynthetic electron transport chain activate the apoplastic NADPH oxidases called respiratory burst oxidative homologues (RBOHs), and they are essential for the execution of HR [75]. The localised cell death caused by the non-host hemibiotrophic bacterial pathogen *X. campestris* pv *vesicatoria* in tobacco leaves was inhibited in transgenic lines expressing a plastid-targeted cyanobacterial flavodoxin in which chloroplast ROS accumulation was suppressed. In this non-host interaction, chloroplast ROS were essential for executing localised cell death but did not contribute to other defence responses, such as the induction of PR genes [75]. The contribution of chloroplast-generated ROS in plant–pathogen interactions also depends on the lifestyle of the invading pathogen. Chloroplast ROS were suggested to play an essential role in the virulence of the necrotrophic fungus *B. cinerea* as the flavodoxin-expressing tobacco plants, which accumulate lower chloroplast ROS after infection, showed enhanced resistance against this necrotrophic pathogen, manifested by reduced tissue damage and fungal growth [76]. This is consistent with previous findings indicating that ROS accumulation and HR promote the colonisation of plant tissue by necrotrophic pathogens [77,78].

Hydrogen peroxide generated in peroxisomes induced the expression of genes involved in SA biosynthesis and signalling [53]. In tomato plants, glycolate-oxidase (GOX)-derived H_2_O_2_ contributed to basal defence against *P. syringae* by modulating SA signalling. The production of H_2_O_2_ in GOX2-silenced plants was significantly reduced, corresponding to increased susceptibility to *P. syringae* [79]. The study by Rojas and Mysore [80] on a set of various single and double mutants of *Arabidopsis* indicated that GOX plays a role in nonhost resistance against *P. syringae* independently of NADPH oxidase action. In rice (*Oryza sativa*), GOX production of H_2_O_2_, which is involved in pathogen resistance, was modulated by the physical association–dissociation of GOX with CAT [81]. Another source of H_2_O_2_ in peroxisomes is acyl-CoA oxidase, which participates in fatty acid β-oxidation involved in the biosynthesis of JA [82]. JA is considered a stress phytohormone, conferring resistance to necrotrophic pathogens and interacting antagonistically with SA, which mediates defence against biotrophic and hemibiotrophic pathogens [83,84]. Salicylic acid triggers a redox-regulated transcriptional response mediated by monomerisation of the transcriptional regulator NPR1 (NON-EXPRESSOR OF PATHOGENESIS-RELATED GENES 1). This response includes the activation of PR gene transcription and transcription factors. Conversely, JA counteracts this process by promoting NPR1 oligomerisation [85]. Although NPR1 is also involved in response to abiotic stresses [86], the stress-specific ROS signature affecting the translocation of redox-dependent transcriptional regulators to the nucleus contributes to the specificity of plant defence responses to different stresses.

In some plants, however, SA and JA interact positively. For example, synergistic interaction between JA and SA signalling was crucial for systemic acquired resistance in the *Brassica napus*-*X. campestris* pv *campestris* pathosystem [87]. Possible interactions between different phytohormones during plant defence reactions have been discussed in detail in recent reviews [88,89], but the role of photorespiration-related elements in these regulations requires further clarification.

The concentration of peroxisomal H_2_O_2_ is regulated by matrix catalase (CAT) and membrane-bound APX. Due to the complementary kinetic properties of CAT and APX, the peroxisomal H_2_O_2_ is highly regulated when it performs its signalling function in response to stress [90]. For example, CAT activity increased in tomato (*S. lycopersicum*) leaves infected with *B. cinerea* [29]. At the same time, peroxisomal APX decreased, confirming their involvement in regulating the H_2_O_2_ content in plants responding to biotic stress. On the other hand, *P. sojae* secretes cytoplasmic effectors, which interact directly with CATs and relocate these enzymes from peroxisomes to the nucleus in the infected cells of *N. benthamiana* [91]. By hijacking plant CATs, these effectors interfere with H_2_O_2_ homeostasis to manipulate programmed cell death and promote infections.

The basal level of peroxisomal H_2_O_2_ affects the antioxidative defence regulation in other compartments, such as cytosol and chloroplasts, as shown in rice plants deficient in peroxisomal APX and subjected to CAT inhibition [92]. The relationship between H_2_O_2_ accumulation and organelle communication was supported by electron microscopy images from mesophyll cells showing stress-induced H_2_O_2_ accumulation in peroxisomes in the contact site with chloroplasts and vacuoles [93]. Peroxisomes interact with other organelles by exchanging H_2_O_2_, redox metabolites, and proteins, and, in recent years, peroxisomes have been indicated as important redox junctions in the communication within plant cells and their stress response [82,94]. Hydrogen peroxide generated in chloroplasts and peroxisomes can induce two different responses by acting as a signalling molecule, as shown in a few studies [95]. The peroxisomal H_2_O_2_-induced transcriptional response is specifically dependent on the subcellular production site and related to stress acclimation/tolerance, while chloroplast H_2_O_2_ induces and integrates signalling responses throughout the cell.

The quality and abundance of peroxisomes are regulated, among other things, by pexophagy, the selective autophagic elimination of obsolete and damaged peroxisomes to prevent metabolic and redox disturbances. As pexophagy is regulated by oxidative stress and is especially abundant under biotic stress, it was suggested to be a mechanism required for fine-tuned H_2_O_2_ homeostasis regulation [94]. In addition to the relatively well-known role of H_2_O_2_/ROS in response to biotic stress, the presence of nitric oxide (NO), synthesis, and metabolism of various reactive nitrogen species (RNS) have been repeatedly observed in peroxisomes [94], although the functions of RNS in peroxisomes during microbe infection remain elusive. However, it is worth noting that the photorespiratory organelles are all considered sites of NO production [96] and are linked with NO function. These organelles also generate ROS, and the interplay between ROS and NO/RNS seems important in regulating metabolic and immune-related processes that govern the outcome of plant–pathogen interactions. For example, in tobacco challenged with *P. syringae* pv *phaseolicola*, NO modulated the primary metabolism during the HR [97], which occurs concurrently with the oxidative burst and H_2_O_2_ production. In tomato plants infected with *B. cinerea*, NO was found to accumulate in the chloroplasts. This was suggested to be rather a stress marker than an effective defence response as in plants with *Trichoderma virens*-induced resistance to *B. cinerea* NO accumulated in the apoplast and nuclei but not in the chloroplasts [98].

**Figure 2:**
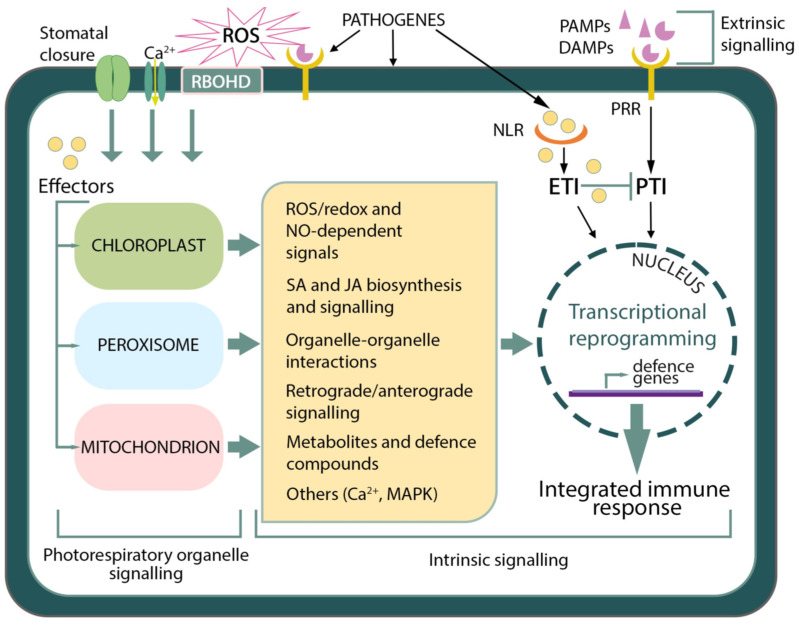
Schematic representation of events associated with photorespiratory organelles during plant–pathogen interactions. Plants perceive pathogen-expressed PAMPs (e.g., flg22) or endogenous elicitors (DAMPs) via extracellular receptors (PRR) and initiate PTI. After PAMP/DAMP perception, a rise in cytosolic Ca^2+^ levels and extracellular ROS production by NADPH-oxidase homolog RBOHD and peroxidases occur. Calcium (Ca^2+^) is involved in regulating apoplast ROS production and intracellular stress signalling. ROS can directly inhibit pathogen growth and influence redox homeostasis in cellular compartments and pathogen-response signal transduction pathways. Pathogen sensing and ROS accumulation in the apoplast lead to stomatal closure, restricting pathogen entry. Stomatal closure triggers increased photorespiratory activity and modulates the status of defence phytohormones such as SA, JA and ABA. Pathogens deliver effectors sensed by intracellular NLR, leading to ETI exemplified by HR. Effectors can suppress PTI and facilitate virulence, e.g., COR promotes stomata reopening by manipulating stress signalling. Pathogen effectors also target chloroplasts, peroxisomes and mitochondria, triggering different molecular mechanisms to inhibit plant immunity. Enhanced photorespiration supports plant immunity. Photorespiratory H_2_O_2_ and redox regulators, metabolites (glycine, serine) and enzymes (GOX, GDC), along with NO and Ca^2+^, may help resist pathogens by limiting microbial growth and upregulating defence gene expression. During photorespiration, the organelle-organelle interactions between chloroplasts, peroxisomes and mitochondria and between them and other cellular compartments (e.g., nucleus, plasma membrane and endoplasmic reticulum) are facilitated by organelle protrusions (stromules, peroxules and matrixules) and membrane contact sites. This enables the exchange of immune signals during ETI. The retrograde/anterograde signalling positions chloroplasts and mitochondria as essential hubs in sensing and integrating various stress signals and regulating metabolism during the response to biotic stress. Abbreviations: ABA—abscisic acid; CO—coronatine; DAMPs—damage-associated molecular patterns; ETI—effector-triggered immunity; GDC—glycine decarboxylase; GOX—glycolate oxidase; HR—hypersensitive response; JA—jasmonic acid; MAPK—mitogen-activated protein kinase; NLRs—nucleotide-binding leucine-rich repeat receptors; NO–nitric oxide; PAMPs—pathogen-associated molecular patterns; PRRs—pattern recognition receptors; PTI—pattern-triggered immunity; RBOHD—respiratory burst oxidase homolog protein D; ROS—reactive oxygen species; SA—salicylic acid.

Chloroplasts, mitochondria, and peroxisomes can control their ROS levels through organelle-specific ROS production, scavenging and transport mechanisms, and the retrograde/anterograde regulations between these organelles and the nucleus. Simultaneously, they can impact the levels of ROS in each other and the nucleus through membrane extensions (e.g., stromules, peroxules, and matrixules), aquaporin-facilitated H_2_O_2_ diffusion, and metabolite/hormone/protein-mediated signalling [99]. Although ROS accumulation in chloroplasts, mitochondria, and peroxisomes occurs during different abiotic and biotic stresses, a stress-specific ROS signature is generated because these organelles are linked to each other, and the ROS transport between them is regulated. Therefore, independent of extrinsic (apoplast and cell wall) ROS signalling, ROS from the chloroplast–mitochondria–peroxisomes network can convey specific information to the nucleus to trigger stress-specific defence mechanisms. These stress-specific ROS/redox-signalling events are interconnected with other signalling pathways, for example, those mediated by phytohormones (Figure 2). Defence-related phytohormones, namely SA, JA, ethylene, and ABA, mediate redox homeostasis by influencing the activities of antioxidant enzymes, such as APX, SOD, and CAT, and the contents of ascorbic acid and glutathione [88].

## 5. Photorespiratory Metabolites and Enzymes in Plant–Pathogen Interactions

Photorespiration plays multiple roles in plant–pathogen interactions beyond just processing reactive oxygen species [53]. Many photorespiratory enzymes are upregulated in infected plants and are involved in the activation of plant defence against pathogens (Table 1). GOX, a crucial photorespiratory enzyme responsible for H_2_O_2_ production in plant cells, is upregulated in response to the fungal pathogens *Leptosphaeria maculans* and *Bipolaris sorokiniana* [100,101]. Accordingly, the silencing of GOX delays the onset of HR in *N. benthamiana*, making the plants susceptible to a range of non-host pathogens [102,103]. Glyoxylate produced by GOX can be further converted into glycine by two glyoxylate aminotransferases in peroxisomes, GGAT, and SGAT. The activities of both aminotransferases were increased in melon (*Cucumis melo*) cultivars resistant to the oomycete pathogen *Pseudoperonospora cubensis* compared with the susceptible ones. A similar trend was observed for GOX, located upstream of these aminotransferases. Elevated activity of the photorespiratory aminotransferases confers pathogen resistance in melon by stimulating GOX and the intraperoxisomal production of H_2_O_2_, activating the immune response. Therefore, resistance was attributed to the activity of GOX that releases H_2_O_2_ [104]. Serine hydroxymethyltransferase (SHMT), important for the cellular one-carbon metabolism catalysing the conversion of serine to glycine in mitochondria, was implicated in HR response in *Arabidopsis* inoculated with avirulent *P. syringae* pv *tomato* DC3000. The *Arabidopsis* mutant *shmt1-1* spontaneously formed lesions and constitutively expressed PR proteins before infection but was more susceptible to biotrophic and necrotrophic pathogens [105]. Collectively, it has been proposed that GOX-generated H_2_O_2_ activates downstream defence responses, leading to HR, while SHMT activity antagonises GOX, repressing downstream defence responses related to the stress phytohormones SA and ethylene [31].

GOX and glyoxylate play a role in diverse biochemical mechanisms [106]. Glyoxylate is typically confined to the peroxisomal matrix. However, under high-photorespiration conditions, a fraction of glyoxylate can be transferred to the cytosol to be reduced to glycolate by the NADPH-dependent glyoxylate/succinic semialdehyde reductase [107]. Glycolate can re-enter peroxisomes and be re-oxidised to glyoxylate, increasing the content of H_2_O_2_, which is an essential signal in the interaction between plants and pathogens [90]. Glyoxylate, the second product of GOX activity, is also involved in plant resistance to pathogens. It is a precursor to oxalic acid, which plays a role in plant interactions with necrotrophic pathogens such as *Sclerotinia sclerotiorum* [108].

GDC, a mitochondrial matrix-localised enzyme responsible for converting glycine to serine, is controlled by NO via S-nitrosylation. As AOX controls mitochondrial NO, this interaction potentially links AOX and photorespiration [109]. Moreover, in wheat, AOX was more strongly activated by intermediates of photorespiration than by pyruvate [110]. AOX contributed to *N. attenuata* resistance to *P. syringae* pv *tomato* DC3000. The infected plants benefited from the activity of AOX and AOX-associated changes in SA and redox levels [111]. GDC was found to be targeted by victorin, a host-selective toxin of the fungal pathogen *Colchiobolus victoriae*, and this interaction was sufficient to cause plant cell death [112].

Photorespiration can generate metabolites, such as glycine, serine, and one-carbon units, for different defence processes (Figure 1). Glycine is mainly provided by photorespiration, and threonine aldolase, an alternative pathway to photorespiration for glycine biosynthesis, can only account for 50% of the amino acid content [113]. Glycine is required for glutathione biosynthesis, and glutathione is a major determinant of cellular redox homeostasis and is essential in plant immunity (for a recent review, see [114]). Plant glycine-rich proteins are involved in plant–pathogen interactions [115]. However, their role is dependent on the pathogen type, and the *Arabidopsis* glycine-rich RNA-binding protein AtGRP7 plays a positive role in defence against necrotrophic bacteria *Pectobacterium carotovorum* and a negative role in response to necrotrophic fungus *B. cinerea* [116]. In the photosynthetic cells of C_3_ plants, serine, mainly produced by photorespiration, reaches a concentration of the order of tens of millimolar, making it the highest amino acid concentration after glutamate and aspartate [117]. Serine is a precursor of sphingolipids and phospholipids, and phospholipid-derived molecules, e.g., oxylipins, are involved in signalling and immune responses during plant–pathogen interactions [118]. Serine and glycine also act as metabolic signals for the transcriptional regulation of the photorespiratory genes encoding enzymes such as GDC and SHMT [119].

Photorespiration is connected with nitrogen and sulphur metabolism, both playing important roles in defence responses to pathogens, and high photorespiratory flux was suggested to be necessary for the assimilation of nitrogen and sulphur [16]. Due to the involvement of nitrogen-containing compounds, i.e., ammonium, glycine, and serine, in photorespiration, this pathway is intimately linked to nitrogen assimilation. Considering plant pathogens, nitrogen can affect their infection strategy and the deployment of virulence factors. In plants, nitrogen mediates biochemical defences, including phytoalexins, antimicrobial proteins, defence-related enzymes, and phytohormones, and regulates defence gene expression through transcription factors or NO. NO plays a signalling role in local defence response and SAR (reviewed in [120]).

Photorespiration may stimulate sulphur assimilation because serine and one-carbon units involved in its photorespiratory production are used to synthesise de novo the sulphur-containing amino acids methionine and cysteine [121]. Cysteine is the primary substrate for the synthesis of glutathione and many sulphur-containing defence compounds, e.g., phytoalexins. Moreover, S-nitrosoglutathione, an NO donor, plays a significant role in NO-related defence signalling and the ROS/RNS interplay in plant immune responses [122]. Cysteine has also been proposed to directly control fungal diseases of grapevine (*Vitis vinifera*) caused by *Phaeomoniella chlamydospore* and *Phaeoacremonium minimum* by inhibiting mycelial growth and spore germination [123].

Plants regulate their defence mechanisms, including PTI, ETI, defence gene expression, and SA- and JA-mediated signalling, via the circadian clock. Accordingly, clock dysfunction in *Arabidopsis* results in compromised resistance to various pathogens [124]. Interestingly, the clock is also linked with photorespiration. CAT activity, a key photorespiratory enzyme regulating H_2_O_2_ homeostasis and signalling, exhibits daily rhythmic variations [125]. The circadian clock also controls the glycine content and SHMT gene expression in *Arabidopsis* [126,127]. As the circadian clock controls defence responses in plants, the circadian interaction with the photorespiratory pathway might play a role in regulating plant immune mechanisms. However, this exciting issue certainly requires further research and explanation.

**Table 1:** Enzymes operating in photorespiration reactions in various plant cell organelles and their involvement in plant–pathogen interactions, on selected examples from recent years of research *.

Enzyme	Organellum	Pathogen	Infected Plant	Infection Effect	References
Glycolate oxidase (GOX, EC 1.1.3.15)	Peroxisome	*Diaporthe citri*	*Citrus reticulata*	Upregulation of four GOX genes	[128,129,130,131,132]
		*Verticillium* *dahliae*	*Gossypium* *hirsutum*	Six GOX gene expression (among 14) changes	
		*Septoria* *lycopersici*	*Solanum* *lycopersicum*	Upregulation of genes: GOX1 and GOX2	
		*Bremia* *lactucae*	*Lactuca sativa*	LsGOX1 and LsGOX2 genes were slightly downregulated	
		*Erwinia* *amylovora*	*Arabidopsis* *thaliana*	GOX activity increased in leaves; gox2-2 mutant sensitive to *E. amylovora*	
Aminotransferases (GGAT EC 2.6.1.4; SGAT EC 2.6.1.45)	Peroxisome	*Botrytis* *cinerea*	*Arabidopsis* *thaliana*	GGAT1 participate in the negative regulation of the plant systemic resistance against *B. cinerea*	[133]
Glycine decarboxylase (GDC, EC 2.1.2.10)	Mitochondrion	*Phytophthora* *infestans*	*Solanum* *tuberosum*	Absence of GDC protein after infection	[134,135]
		*Rhizoctonia solani*	*Oryza sativa*	H-protein from glycine decarboxylase complex detected	
Serine hydroxymethyl-transferase (SHMT, EC 2.1.2.1)	Mitochondrion	*Pseudomonas* *syringae* pv *tomato*	*Arabidopsis* *thaliana*	SHMT6 overexpression lines —enhanced resistance	[132,135,136]
		*Septoria* *lycopersici*	*Solanum* *lycopersicum*	Upregulation of genes: SHMT2, SMHT3	
		*Phytophthora* *infestans*	*Solanum* *tuberosum*	Absence of SHMT protein after infection	
Glycerate-3-kinase (GLYK, EC 2.7.1.31)	Chloroplast	*Septoria* *lycopersici*	*Solanum* *lycopersicum*	GLYK gene upregulation	[132]

* older papers are cited in [32].

## 6. Mitochondria and Respiration Under Biotic Stress—Various Aspects in a Nutshell

Previous studies have repeatedly shown an increase in the intensity of cellular respiration and increased ATP synthesis in the initial period after infection by pathogens to provide carbon skeletons and energy for defence-related metabolite production (reviewed by [137]). Recent decades of research have brought discoveries regarding the functions of mitochondria, going far beyond only the production of ATP in the respiratory process. One of the exciting discoveries was the demonstration of the participation of these organelles in the immune response, both in animal cells, where the mechanism of mitochondria-mediated signalling was proposed first [138,139], and in plant cells [140,141]. ROS are produced in mitochondria, as in peroxisomes and chloroplasts, and ROS are proposed as the key signals that initiate defence reactions [138,141,142]. The latest works point, in particular, to several aspects of the participation of plant mitochondria in plant immunity. These are, for example, (i) mitochondrial permeability transition pores, including membrane voltage-dependent anion channels (VDACs) participating in the onset of H_2_O_2_-mediated programmed cell death (PCD); (ii) mitochondrial dynamics—size–shape modifications, or cell position changes, in response to pathogen invasion; (iii) involvement of numerous components of mitochondrial metabolic processes, e.g., AOX in defence response; (iv) function of mitochondria in SA-mediated defence (reviewed by [141]). In addition, the results of Khan et al. [142] suggest that ROS generated in mitochondria initiate mitochondrial retrograde regulation, even without leaking into other plant cell compartments. Mitochondria are also targeted by pathogen effectors suppressing host defence. For example, the type III effector HopG1 secreted by the hemibiotrophic bacterial pathogen *P. syringae* pv *tomato* localises to mitochondria, disturbs mitochondrial respiration, and suppresses PTI and ETI [143]. The necrotrophic fungus *S. sclerotiorum* secretes the proteinaceous effector SsSSVP1, which interacts with a subunit of the cytochrome b-c1 complex in the mitochondrial respiratory chain [144]. This interaction disturbs its subcellular localisation, manipulating plant energy metabolism and increasing ROS production and PCD to facilitate infection.

Several recent reviews have discussed mitochondrial roles in plant stress responses in detail [32,109,140,141,145,146]. Thus, we focus on the role of selected components of the respiratory pathway in plant response to pathogens, emphasising the alternative pathway in the cell. AOX, a small enzymatic protein located in the inner membrane of mitochondria, usually encoded by several genes, is a crucial enzyme in the alternative cyanide-resistant respiratory pathway. AOX determines the direct transfer of electrons from ubiquinone to oxygen in the mitochondrial respiratory chain; the enzyme activity reduces the energy efficiency/ATP production during respiration. It is regulated at the transcriptional and post-translational levels by stress factors, including infection by pathogens [109,147,148,149]. AOX is involved in adaptation to various stresses, participating in energy and redox balance regulation in mitochondria and the plant cell [109,150,151]. The influence of AOX on redox balance is not restricted to ROS; its interaction with NO implicated in defence against pathogens is also important [5]. AOX can interact with stress signalling via ROS generation and cell energy status modifications, often cooperating with alternative NAD(P)H dehydrogenases. Recent studies indicated that AOX also coordinates the expression of other genes encoding proteins acting in the electron transport chain and could generate or amplify some stress signals, including those in HR [148,150]. However, the exact role of AOX under biotic stress conditions is currently less known than in the case of abiotic stresses; let us hope that, soon, new studies will bring the expected progress in this area of knowledge.

## 7. Conclusions and Future Perspectives

Strategies for coping with pathogen infections regulate major adaptations in plant primary metabolism. Over the past few decades, there has been considerable progress in understanding the role of photorespiration-related processes in plant defence responses. As summarised in Figure 2, photorespiration plays important and multiple roles in plant–pathogen interactions. However, there are still significant gaps in our knowledge, particularly concerning the mechanisms and physiological functions of photorespiratory enzymes and metabolites in crop plants under pathogen-related stress. Individual plants show considerable variation in their responses to pathogens, likely due to differences in experimental conditions and the specific host–pathogen systems used in the laboratory. Furthermore, findings from laboratory studies do not always translate to more complex natural conditions, where multiple biotic and abiotic stresses may co-occur. Although there are some commonalities in responses to abiotic and biotic stresses, the magnitude and kinetics of photorespiration-related immune signalling could shape the specificity of PTI and ETI during host–pathogen interactions. More extensive research is needed to unravel the post-infectious inter-organellar communication and transport routes of various compounds, including signalling molecules, between photorespiratory organelles. With modern genetic, proteomic, and metabolomic approaches and techniques, we hope to better regulate the relationship between photorespiration and photosynthesis in the future. This could lead to developing crop plants that exhibit optimal growth and enhanced photosynthetic productivity, ensuring food security amid a changing climate and a growing global population.

## Data Availability

Not applicable.
